# Molecular evolution of the capsid gene in human norovirus genogroup II

**DOI:** 10.1038/srep29400

**Published:** 2016-07-07

**Authors:** Miho Kobayashi, Yuki Matsushima, Takumi Motoya, Naomi Sakon, Naoki Shigemoto, Reiko Okamoto-Nakagawa, Koichi Nishimura, Yasutaka Yamashita, Makoto Kuroda, Nobuhiro Saruki, Akihide Ryo, Takeshi Saraya, Yukio Morita, Komei Shirabe, Mariko Ishikawa, Tomoko Takahashi, Hiroto Shinomiya, Nobuhiko Okabe, Koo Nagasawa, Yoshiyuki Suzuki, Kazuhiko Katayama, Hirokazu Kimura

**Affiliations:** 1 Gunma Prefectural Institute of Public Health and Environmental Science, Maebashi-shi, Gunma 371-0052, Japan; 2 Kawasaki City Institute for Public Health, Kawasaki-shi, Kanagawa 210-0821, Japan; 3 Ibaraki Prefectural Institute of Public Health, Mito-shi, Ibaraki 310-0852, Japan; 4 Osaka Prefectural Institute of Public Health, Osaka-shi, Osaka 537-0025, Japan; 5 Hiroshima Prefectural Technology Research Institute, Public Health and Environment Center, Hiroshima-shi, Hiroshima 734-0007, Japan; 6 Yamaguchi Prefectural Institute of Public Health and Environment, Yamaguchi-shi, Yamaguchi 753-0821, Japan; 7 Kumamoto Prefectural Institute of Public-Health and Environmental Science, Uto-shi, Kumamoto 869-0425, Japan; 8 Ehime Prefectural Institute of Public Health and Environmental Science, Matsuyama-shi, Ehime 790-0003, Japan; 9 Pathogen Genomics Center, National Institute of Infectious Diseases, Musashimurayama-shi, Tokyo 208-0011, Japan; 10 Department of Microbiology, Yokohama City University Graduate School of Medicine, Yokohama-shi, Kanagawa 236-0027, Japan; 11 Department of 1st Internal Medicine, Kyorin University School of Medicine, Mitaka-shi, Tokyo 181-0004, Japan; 12 Department of Food and Nutrition, Tokyo Kasei University, Itabashi-ku, Tokyo 173-0003, Japan; 13 Iwate Prefectural Meat Inspection Center, Shiwa-cho, Iwate 020-3311, Japan; 14 Infectious Disease Surveillance Center, National Institute of Infectious Diseases, Musashimurayama-shi, Tokyo 208-0011, Japan; 15 Division of Biological Science, Nagoya City University, Nagoya-shi, Aichi 467-0000, Japan; 16 Department of Virology II, National Institute of Infectious Diseases, Musashimurayama-shi, Tokyo 208-0011, Japan

## Abstract

Capsid protein of norovirus genogroup II (GII) plays crucial roles in host infection. Although studies on capsid gene evolution have been conducted for a few genotypes of norovirus, the molecular evolution of norovirus GII is not well understood. Here we report the molecular evolution of all GII genotypes, using various bioinformatics techniques. The time-scaled phylogenetic tree showed that the present GII strains diverged from GIV around 1630CE at a high evolutionary rate (around 10^−3^ substitutions/site/year), resulting in three lineages. The GII capsid gene had large pairwise distances (maximum > 0.39). The effective population sizes of the present GII strains were large (>10^2^) for about 400 years. Positive (20) and negative (over 450) selection sites were estimated. Moreover, some linear and conformational B-cell epitopes were found in the deduced GII capsid protein. These results suggested that norovirus GII strains rapidly evolved with high divergence and adaptation to humans.

Norovirus (NoV) is a pathogenic agent of acute gastroenteritis in humans^1^. It has led to pandemics of acute gastroenteritis around the world^1^. In Japan, half of acute gastroenteritis cases in the winter season may be caused by NoV infection^2^,^3^. Furthermore, large outbreaks of food poisoning involving NoV have been reported in many countries^4^,^5^. Thus, NoV is a major causative agent of acute viral gastroenteritis worldwide, and NoV infection is a major disease burden in many countries^1^,^6^.

NoV belongs to the genus *Norovirus* and the family *Caliciviridae* and, at present, is classified into seven genogroups (GI–GVII), based on phylogenetic analysis of the capsid gene^7^. Among them, NoV belonging to genogroups I, II, and IV may infect humans^7^. Furthermore, the NoV GI and GII strains can be classified into 9 and 22 genotypes, respectively^8^.

Previous epidemiological studies suggested that specific genogroup/genotype viruses (e.g., GII.2, GII.3, GII.4, and GII.6) caused more recent large outbreaks of gastroenteritis than other GII and GI genotypes^9^,^10^,^11^. In particular, endemics of gastroenteritis caused by GII.4 have been recognized for at least 20 years^12^,^13^,^14^. Furthermore, another genotype, GII.P17-GII.17 virus, emerged in 2013 and spread rapidly as GII.4^15^.

To gain a better understanding of antigenic variations in the molecular evolution of NoV, it is essential to analyze the capsid gene. The capsid protein, encoded by the second of three open reading frames^1^, is crucial for viral adsorption and entry and the production of neutralizing antibodies^16^,^17^,^18^,^19^. Thus, predicting the common epitopes in the capsid protein (major antigen) may aid the development of an effective vaccine against NoV.

Recently, various bioinformatics technologies have enabled estimations of the phylogenies and genetic properties of diverse viruses, including NoV^20^,^21^. For example, the Bayesian Markov Chain Monte Carlo (MCMC) method was used to estimate the evolutionary time-scale of the capsid gene in NoV GI^22^. Siebenga *et al*. and Eden *et al*. reported the molecular evolution of GII.4^20^,^21^. Furthermore, *in silico* methods may be able to predict the linear and conformational epitopes in the antigens of NoV^23^. Studies on the molecular evolution of NoV GII have been performed in part for some genotypes^20^,^21^. However, NoV GI and GII are genetically quite different, although they are classified in the same family and genus^1^,^8^. Moreover, a detailed understanding of the molecular evolution of the capsid gene is an open issue. Therefore, in the present study, we conducted a comprehensive study into the molecular evolution of the capsid gene for all GII genotype strains, using bioinformatics algorithms similar to a previous work^22^.

## Results

### Phylogenetic analysis of NoV capsid gene using Bayesian Markov chain Monte Carlo methods

**Figure 1:**
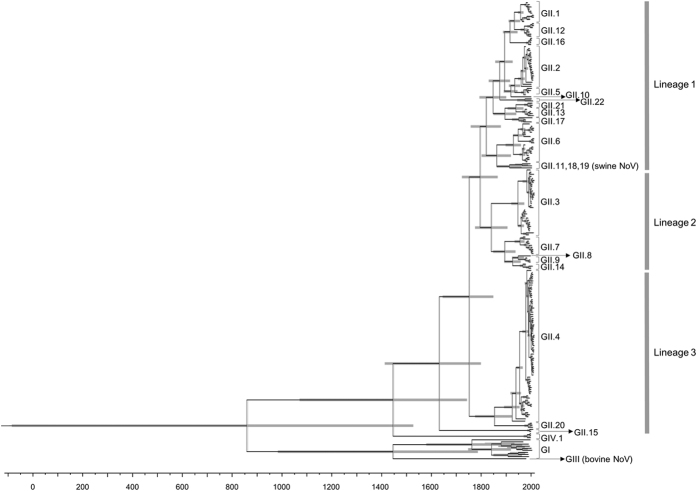
Phylogenetic tree of the capsid gene on NoV constructed by the Bayesian MCMC method. 203 strains of human GII, three strains of swine GII, nine strains of GI, one strain of GIII, and three strains of GIV were included in this tree. Grey bars show 95% HPDs. The scale bar represents actual time (year). The time of the most recent common ancestor of this tree was around 854 CE. GII strains were divided from GIV around 1630 CE. NoV GII was formed three lineages.

We constructed a phylogenetic tree, based on the capsid gene by the Bayesian MCMC method (Fig. 1). To gain an understanding of the time scale of the phylogeny of the full-length capsid gene, we used 206 strains of all genotypes of NoV GII (22 genotypes) and 13 strains of other genogroups/genotypes (total 219 strains).

First, the MCMC phylogenetic tree showed that the 22 genotypes of NoV GII strains could be classified into three lineages: lineage 1 (GII.1, 2, 5, 6, 10–13, 16–19, 21 and 22), lineage 2 (GII.3, 7, 8, 9 and 14), and lineage 3 (GII.4, 15 and 20; Fig. 1). Each lineage contained one or two major genotypes (lineage 1, GII.2 and GII.6; lineage 2, GII.3; and lineage 3, GII.4).

Next, the MCMC tree showed that the most recent common ancestor of the tree was around 854 CE (95% highest posterior densities [HPDs] 53 BCE–1537 CE; Fig. 1). The ancestor of the GII strain diverged around 1630 CE (95% HPDs 1409–1796 CE). Three major lineages and the common ancestor of GIV date back to around 1445 CE (95% HPDs 1065–1739 CE). The years of divergence of each lineage, genotype, and genogroup are presented in Supplementary Table S1. Lineage 3 diverged in 1630 CE, lineage 1 in 1819 CE, and lineage 2 in 1839 CE (Fig. 1 and Supplementary Table S1). The mean evolutionary rate of the present human GII strains was estimated to be 3.76 × 10^−3^ substitutions/site/year (95% HPDs 3.21 × 10^−3^–4.30 × 10^−3^ substitutions/site/year). The results suggested that the present GII strains formed three major lineages at a high evolutionary rate (around 10^−3^ substitutions/site/year) and the common ancestor dates back over 500 years.

### Pairwise distances (*p*-distances) among genogroups and lineages

We analyzed the distribution of *p*-distances among the present strains (Supplementary Fig. S1a–d). Human NoV GII had a large *p*-distance (mean ± standard deviation [SD]; 0.286 ± 0.094), based on the nucleotide sequences of the capsid gene (Supplementary Fig. S1a). The maximum pairwise distance was 0.398. The *p*-distance values of lineages 1, 2, and 3 were 0.283 ± 0.081 (mean ± SD), 0.205 ± 0.117, and 0.119 ± 0.089, respectively (Supplementary Fig. S1b–d). The results suggested that the capsid gene of NoV GII has a high degree of genetic divergence.

### Phylodynamics of human NoV GII strains

**Figure 2:**
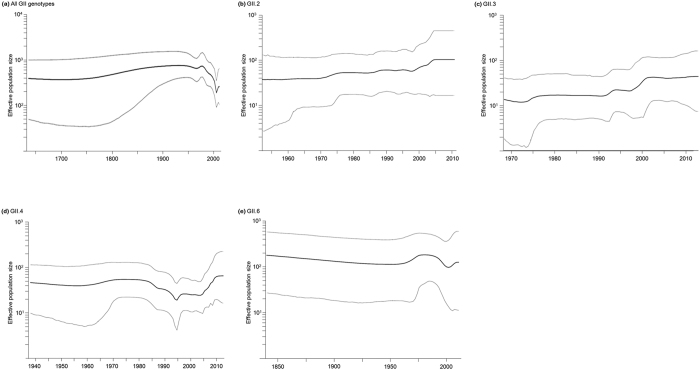
Bayesian skyline plots of all NoV GII (**a**) GII.2 (**b**) GII.3 (**c**) GII.4 (**d**) and GII.6 (**e**). The x-axis represents actual time (years) and starts at mean tree model root height. The y-axis represents the effective population size. Mean effective population size is shown as a black line. HPDs of 95% are shown as grey lines.

We estimated the effective population sizes of the capsid gene of human NoV GII strains in Bayesian skyline plots (BSPs; Fig. 2a). In the present human NoV GII strains, the mean effective population size remained constant until the 1960s. Thereafter, it decreased temporally and increased again around 2000 CE. We also performed BSP analysis of the major prevalent genotypes, such as GII.2, 3, 4, and 6^9^,^10^,^11^. Although the mean effective population sizes of GII.2 and GII.3 grew slowly after the 1970s, those of GII.4 and GII.6 remained unstable throughout the plotted times (1937–2013 for GII.4, 1839–2012 for GII.6) (Fig. 2b–e). Notably, the effective population sizes of GII.4 declined from the 1980s to the middle of the 1990s, but these values increased during the past 15 years (Fig. 2d). The GII.6 values reached a small peak around 1990 and decreased slightly thereafter (Fig. 2e). The GII.2 and GII.3 values increased slightly after 2000 (Fig. 2b,c), and the GII.6 values increased in the 1970/80s and decreased thereafter (Fig. 2e). Overall, the effective population sizes of all NoV GII strains were estimated to be 10^2^ for about 400 years. The results suggested that NoV GII strains have become highly adapted to humans over a long period.

### Estimation of positive selection sites and negative selection sites in human NoV GII

The selection pressures on each site in the capsid gene were analyzed for the present GII strains. Positively selected sites were estimated by four methods: single likelihood ancestor counting (SLAC), fixed effects likelihood (FEL), internal fixed effects likelihood (IFEL), and mixed effects model of evolution (MEME)^24^,^25^; 20 sites under positive selection were detected (Table 1). Common sites under positive selection estimated by the four methods occurred after amino acid changes at two sites: Ser6Asn and Asn6Ser/Lys/Ile and Arg435Thr/His, Thr435Pro/Val, Pro435His/Ser, His435Ala/Arg/Gln, Ala435Arg/Ser/His/Val, and Gln435Pro. The mean *d*N/*d*S ratio (0.106) obtained by the SLAC method was relatively low (95% confidential intervals; 0.103–0.109). We also detected 489, 498, and 460 sites under negative selection by the SLAC, FEL, and IFEL methods, respectively.

**Figure 3:**
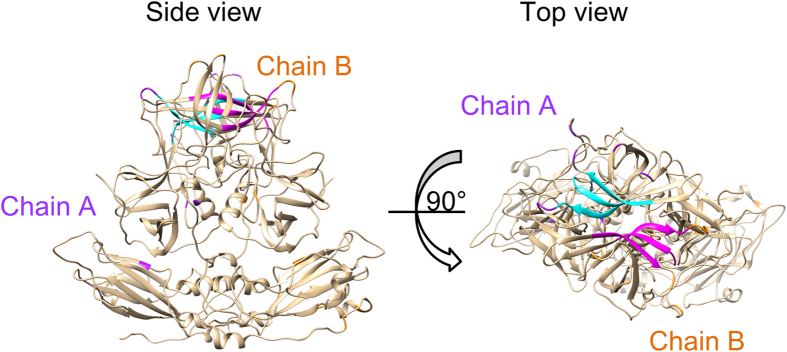
Location of positive selection sites on predicted structure of capsid protein in GII.4/Bristol/1993/UK. To construct the model, we used five suitable templates of NoV capsid sequences (PDB ID: 1IHM, 3ONU, 4RLZ, 3PUM, and 4X07). Twenty positively selected sites on chains A and B are colored purple and orange, respectively. The HBGA binding sites^45^ are colored blue and pink. These sites were located within the surface of the protein.

Furthermore, we mapped the 20 positively selected sites in Table 1 in purple and orange on the dimer of the capsid protein (Fig. 3 and Supplementary Fig. S2). Most of the sites were located within the surface of the capsid protein. The results suggested that selective pressure from host causes amino acid substitution of the virus.

### Epitopes predicted on the deduced capsid protein in human NoV GII

Previous reports studied B-cell epitope predictions with two distinct definitions: linear and conformational epitopes^26^,^27^,^28^,^29^,^30^,^31^,^32^. In this study, we predicted both linear and conformational epitopes of the capsid protein (VP1) in the standard strains of each genotype. Linear epitopes were predicted by combination analysis with seven tools: LEPS^26^, Epitopia^27^, BCPRED^28^, FBCPRED^28^, Bepipred^29^, Antigenic^30^, and LBtope^31^, according to a previous report^33^. GII.6 and GII.12 could not be analyzed. The protein sequences of GII.6 (accession No. AJ277620) and GII.12 (accession No. AJ277618) have unknown amino acids (X) because of including mixed nucleotide sequences.

**Figure 4:**
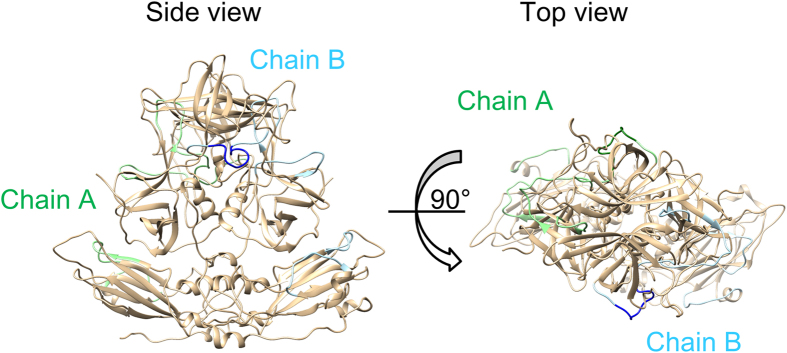
Predicted linear B-cell epitopes mapping on the capsid protein of GII. 4. The predicted structure of capsid protein is the same as in Fig. 3. Linear B-cell epitopes on chain A and B are shown in green and blue, respectively. Common locations among all genotypes are represented by deeper tones. These sites consist of 11 amino acids (DPTXXXPAPXG or similar sequence to this).

The linear epitopes predicted are shown in Table 2. Notably, a common sequence of 11 amino acids (DPTXXXPAPXG or similar sequence to this) was found in almost all GII genotypes, apart from GII.6 and GII.12. The common epitope motif was located in the protruding 2 (P2) domain, which corresponds to the positions at amino acids (aa) 312–322 in the capsid protein of GII.4/Bristol/1993/UK strain. Figure 4 and Supplementary Fig. S3 show the common linear epitopes on the predicted capsid protein structure (dimer) in green and blue.

**Figure 5:**
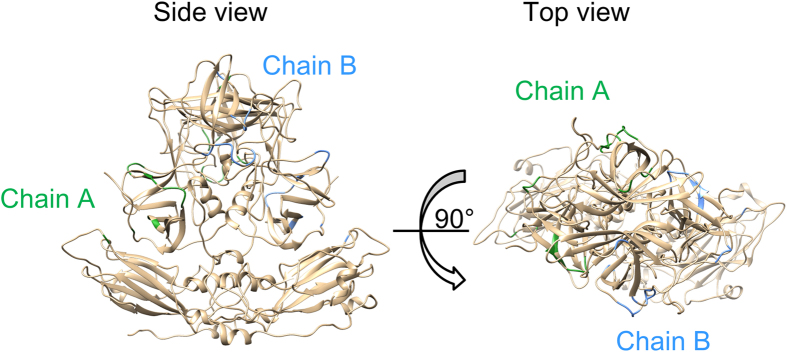
Predicted conformational B-cell epitopes mapping on the capsid protein of GII. 4. The predicted structure of capsid protein is the same as in Fig. 3. These sites on chain A and B are shown in green and blue, respectively. Most of conformational epitopes were located in the P1 and P2 domains.

Next, we predicted the conformational epitopes using CBtope^32^. For each genotype, 4–36 sites were estimated to be conformational epitopes (Supplementary Table S2). The epitopes were mainly located in the P1 and P2 domains on the capsid protein (Fig. 5 and Supplementary Fig. S4).

## Discussion

We completed a comprehensive study on the molecular evolution of the capsid gene in all genotypes of NoV (GII). As a result, we estimated that the common ancestor of the present GII strains diverged from a GIV strain with a high evolutionary rate (around 10^−3^ substitutions/site/year) around 1630 CE and formed three major lineages. The capsid gene in the present GII strains shows a high level of divergence (maximum *p*-distance >0.39). Furthermore, some significant findings were made. 1) The effective population sizes of the present GII strains were relatively large (over 10^2^) during 400 years. 2) Some positive (20 sites) and many negative (over 450 sites) selection sites were estimated. 3) Some linear and conformational B-cell epitopes were found in the predicted capsid protein of GII.

The results suggest that NoV GII strains rapidly evolved with high levels of genetic divergence and adaptation to humans. However, since we obtained the GII capsid gene sequences from GenBank alone, the present data may be subject to selection bias. In addition, the present alignment data of the nucleotide sequences may have a sequence length bias, because these strains belonging to various genogroups show the different nucleotide lengths of the capsid genes. This may reflect on the accuracy of the data. Thus, the bias may limit the present study.

We conducted phylogenetic analyses by the Bayesian MCMC method. The results showed that GII strains formed three major lineages and 22 genotypes with high genetic divergence (Fig. 1). Moreover, the MCMC tree estimated that the common ancestor GII diverged from another genogroup, GIV, about 380 years ago (1630 CE; Fig. 1 and Supplementary Table S1). Thereafter, the present GII strains formed 22 genotypes (Fig. 1). Previous studies reported the molecular evolution of some genotypes/genogroups of NoV^20^,^22^,^34^. For example, Kobayashi *et al*. showed that the evolutionary rate of the GI was estimated as 1.26 × 10^−3^ substitutions/site/years, and GI strains divided into two lineages about 750 years ago^22^. Siebenga *et al*.^20^ estimated the most recently common ancestor year of GII.4 as 1982. Rackoff *et al*.^34^ reported that the evolutionary rate of GI.3 NoV was 1.25 × 10^−3^ substitutions/site/year. Furthermore, other ssRNA virus, such as HIV or H3N2 influenza virus, evolved with similar evolutionary rates of about 10^−3^ substations/sites/year^35^,^36^. In this study, we found that the evolutionary rate of the GII capsid gene was as rapid as that of the GI capsid gene^22^. To our knowledge, these are first descriptions of the evolution of the all genotypes of GII capsid gene.

Our previous study suggested that human NoV GI also had high genetic divergence (maximum *p*-distance values >0.39). The present MCMC tree suggested that all genogroups of NoV have high genetic divergence. These findings may, therefore, indicate the biological divergence of capsid function and host specific infectivity.

Next, the effective population size may reflect virus genome populations in the host during the periods analysed^37^. The effective population size of the present NoV GII strains was relatively large (over 10^2^) for 350 years (Fig. 2a). Our previous study indicated that NoV GI had a large effective population size (about 10^3^) for 500 years^22^. Therefore, like the NoV GI strains, GII strains have become highly adapted to humans because of the effects of natural selection rather than genetic drift. We analyzed the BSP of the major prevalent genotypes, including GII.2, GII.3, GII.4, and GII.6 (Fig. 2b–e). Previous molecular epidemiological reports suggested that these genotypes appeared within the last 20 years^9^,^10^,^11^. Among them, GII.4 is the most dominant^9^,^10^,^11^. Specifically, this genotype has been detected in patients with acute gastroenteritis in various countries since the 1990s^12^,^13^,^14^. Some variants of GII.4 emerged and spread around these countries^1^,^12^,^13^,^14^,^20^,^21^. The BSP data from the present study show that the effective population size of GII.4 increased since 2000 (Fig. 2d). The periods of increased effective population size were preceded by periods of prevalence; such fluctuations in BSP data may help predict the prevalence of NoV. However, we did not exactly examine these relationships among the genogroups, because the data are scarce at present^9^,^10^,^11^. Hence, further and larger studies of each genotype and predictions of their prevalence may be needed.

Host defense mechanisms may affect viral antigens and lead to virus escape mutations^38^. Such substitutions are thought to represent positive selection^38^. In the present GII strains, positive selection was estimated at 20 sites of amino acid substitutions, though the SLAC method estimated two sites (Table 1). The sites under positive selection were mainly located in the P2 domain. In our previous study of NoV GI capsid gene evolution, 19 sites under positive selection were estimated by the MEME method, and no sites were estimated, by the SLAC method, even in the P2 domain^22^. The SLAC method is appropriate for detecting non-neutral evolution^24^ and may be a stricter algorithmic model for estimating positive selection sites. On the other hand, the MEME method considers lineage-to-lineage variations by a nonsynonymous (*d*N) and synonymous (*d*S) substitutions ratio (*d*N/*d*S)^25^. This method is suitable for estimating episodic selective pressure^25^. Thus, the difference of the algorithm reflected the numbers of positive selection sites in the present GII strains. Together, host defence mechanisms and immunity are more effective against the GII capsid protein. The antigenicity of the GII strains may be stronger than that of the GI capsid protein, because the capsid protein in the P2 domain may largely reflect the antigenicity of NoV^1^,^17^.

In the present study, over 450 sites under negative selection were confirmed in the NoV GII capsid protein. Mahar *et al*.^39^ reported many sites under negative selection in the GII capsid protein. Moreover, our previous data showed a large number (over 400 sites) in NoV GI capsid protein, although the locations of the sites under negative selection were different^22^. Negative selection may rephrase stabilising selection^38^. This type of selection may act to eliminate variant genomes, leading to adaptation to an environment, because most of these mutations are deleterious^38^. Thus, negative selection in the present GII strains may prevent deteriorations of capsid protein functions, including infectivity. Furthermore, it may be important to clarify the roles of the negative selections in NoV capsid proteins, although numerous codon substitutions as negative selection sites are inferred in the NoV GII capsid protein. However, regarding each substitution, it may be difficult to computationally and experimentally examine the stability and folding of NoV capsid protein.

In this study, we used four methods (i.e., FEL, IFEL, SLAC, and MEME) to make a candidate list of positively and negatively selected amino acid sites. Based on these analyses, we showed that the biological significance of these sites was validated with the structural data. However, these methods may have advantages and disadvantages^40^. Thus, further and larger studies, including the fitting of the bioinformatics technology, may be needed to understand the roles of the negative selection in the capsid protein.

In addition, we predicted both linear and conformational B-cell epitopes in the capsid protein in GII for all genotype strains. Some epitopes were confirmed for each genotype strain (Table 2 and Supplementary Table S2) by both methods. First, the common location of linear epitopes, apart from GII.6 and GII.12, were confirmed, and the common motif was DPTXXXPAPXG in GII.1, 4, 8, 10, 13, 14, 16, 17, 21, and 22 (Table 2), located at the side of the P2 domain as shown in a deeper tone (Fig. 4 and Supplementary Fig. S3). Moreover, some conformational epitopes were confirmed in each genotype (Supplementary Table S2). Most of the predicted epitopes, however, did not overlap with the blockade epitopes A, D, and E amino acid residues and locations of the capsid protein that predicted with GII.4 NoV^41^ (Fig. 5 and Supplementary Fig. S4). In particular, the common motif DPTXXXPAPXG may not relate to blocking of the HBGA binding. However, it may have an important function that is related to an internalising receptor binding because it is highly conserved among the NoV genotypes.

Previous studies suggest that different NoV genotype strains infect humans^42^. Furthermore, humoral immunity against NoV may not persist for long^42^. Thus, the protective (neutralising) antibodies against the common epitopes in NoV GII strains may not be produced in the host. Alternatively, if antibodies against the common epitopes are produced, they cannot prevent NoV infection of host cells. Further studies on common epitopes in NoV are needed.

Next, histo-blood group antigens (HBGAs) in the host cells may be associated with the binding of NoV GII capsid protein to the P2 domain^43^, and this association may be important for viral attachment to host cells^44^. For example, Cao *et al*.^45^ showed that aa336, aa345, and aa374 in the P2 domain of GII.4/VA387/1998/US strain could bind HBGA, and these were associated with NoV GII infections in the host. Furthermore, host defence mechanisms (i.e., humoral immunity) produce protective antibodies against NoV. If amino acid substitutions occur around HBGA binding sites, the antibodies that block HBGA binding cannot protect the host efficiently against NoV infection^42^. Amino acid substitutions under positive selection were observed at residues 370 and 397, adjacent to the HBGA binding sites (Table 1). In addition, B-cell epitopes may be associated with sites under positive selection^46^. Thus, these substitutions might protect against host immunity.

In conclusion, the common ancestor of GII diverged from GIV around 1630 CE at a high evolutionary rate. The GII capsid gene had very high divergence. In addition, the effective population sizes of GII strains had relatively large values during a prolonged period. NoV GII may have been affected by natural selection and strong selective pressure from the host and may have adapted to humans through these evolutionary processes affecting the capsid gene. These results will be a basis of prediction of escape mutants or novel genotype. While our data should be helpful for developing vaccines or for preventing epidemics, further study is needed.

## Methods

### Strains used in this study

We obtained a comprehensive range of the full-length nucleotide sequences (1620 nt for GII.4/Bristol/1993/UK, Genbank accession No. X76716) of human NoV GII capsid gene, excluding ORF1/2 recombinant strains from GenBank in August 2014. A total of 1582 strains were obtained, and the year in which they were detected was clearly described. These sequences were aligned by Clustal W2^47^. Strains with more than 97.5% identity were excluded from the dataset. Ultimately, 203 strains were used in this study. The average nucleotide divergence in the dataset was 0.54.

### Phylogenetic tree constructed by Bayesian MCMC method

We used Bayesian MCMC method in BEAST package v1.8.2 to estimate the time-scaled phylogenies^48^. To estimate the ancestor of various genogroups of NoV, we added 13 outgroups of NoV, including NoV GI (human type), GII (porcine type), GIII (bovine type), and GIV (human type). Detailed data of the strains are shown in Supplementary Table S3.

First, the substitution model was selected using KAKUSAN 4^49^ with GTR-Γ model. Next, three clock models (strict clock, uncorrelated lognormal relaxed clock, and uncorrelated exponential relaxed clock) and four demographic models (constant size, exponential growth, expansion growth, and logistic growth) were calculated by generating 100,000,000 steps with sampling every 20,000 steps. These models were compared by Akaike’s Information Criterion through MCMC (AICM) using Tracer^50^,^51^. The lowest AICM value was used. Finally, 219 strains were analysed using exponential clock and exponential growth models with coalescent tree prior. The MCMC chain length was 500,000,000 steps with sampling every 20,000 steps. Convergence was evaluated by the effective sample size by Tracer^51^, and values more than 200 were acceptable. The maximum clade credibility tree was obtained after 10% burn-in using TreeAnnotator v1.8.2^48^. The MCMC phylogenetic tree was constructed by FigTree v 1.4.0^48^. The reliability of branches is supported by 95% HPDs.

The evolutionary rate of human NoV GII was also estimated. In this calculation, 203 strains were tested under the best-fit model (GTR-Γ + lognormal relaxed clock + constant size). The MCMC chain length was set at 100,000,000 steps with sampling every 20,000 steps.

### Calculation of pairwise distance (*p*-distance)

We analyzed *p***-**distances to assess the genetic distances between human GII strains. The *p*-distance values of intergenogroup and interlineages were calculated using MEGA 6.0^52^.

### Bayesian skyline plot analysis

BSP analysis was performed to estimate the phylodynamics in human GII strains. Human GII (203 strains) were analysed with the BSP coalescent prior using BEAST v1.8.2^48^. The substitution and clock models were selected using AICM, as mentioned earlier. Datasets were analysed using a GTR-Γ exponential clock model. MCMC chains were run for 1,000,000,000 steps with sampling every 20,000 steps. BSP was constructed using Tracer^51^. We also estimated the effective population sizes of the major genotypes such as GII.2, 3, 4, and 6. Calculations of these genotypes were performed as described earlier. The detailed conditions of analysis are shown in Supplementary Table S4.

### Selective pressure analysis

To find candidates of positive/negative selected sites in capsid protein on human NoV GII, nonsynonymous (*d*N) and synonymous (*d*S) substitutions rates at every codon were calculated using Datamonkey^24^. To multilaterally analyze the selective pressure of NoV capsid gene, we used the following four methods: SLAC, FEL, IFEL, and MEME. SLAC, the fastest method, is appropriate for large (>50) datasets^40^. FEL and IFEL are suitable for intermediate alignments^40^. FEL method directly estimates site-by-site substitutions^40^. Although IFEL method is similar to FEL, it only calculates along the internal branches of the tree^40^. SLAC, FEL and IFEL may appear to underestimate the number of positive selectionsites^25^. MEME method is suitable for estimating episodic positive selections at each site^25^. Sites under positive selection (*d*N > *d*S) were determined by a *p*-value of <0.05. We also estimated negative selection sites (*d*N < *d*S) using SLAC, FEL, and IFEL methods. The *d*N/*d*S ratio was estimated under the MG94 model in the Datamonkey. The cut off *p*-value was at 0.05.

### B-cell epitope prediction of human NoV GII

We predicted both linear and conformational epitopes in the capsid protein, using the deduced amino acid sequences of the standard strains of each genotype. Linear B-cell epitopes were predicted using the following seven tools: LEPS^26^, Epitopia^27^, BCPRED^28^, FBCPRED^28^, BepiPred^29^, Antigenic^30^, and LBtope^31^. These tools were used in default conditions and amino acids estimated by four or more tools with >10 consecutive sites were considered linear B-cell epitopes^33^. In addition, conformational epitopes were predicted using CBtope^32^. The threshold of the support vector machine score was set at 0.0.

### Mapping of positive selection sites and predicted epitopes

A structural model of the standard strains in each genotype was predicted using MODELLER v9.15^53^. Homology modelling was based on the crystal structure of five strains (PDB ID: 1IHM, 3ONU, 4RLZ, 3PUM and 4X07). The capsid structure of GI (PDB ID: 1IHM) was used to construct the whole structure of the VP1 dimer, including the P1 and shell domains. The structures of five templates and the standard strains were aligned by MAFFTash^54^,^55^. To surely provide the structures, the sequence identities of templates and targets were 45.3–100%^56^. The constructed models were minimized by GROMOS96^57^, implemented in Swiss PDB Viewer v4.1^58^ and evaluated by Ramachandran plots through the RAMPAGE server^59^. Final models were modified and coloured by Chimera v1.10.2^60^. Positive selection sites and linear and conformational epitopes of each genotype were mapped on the structures.

## Additional Information

**How to cite this article**: Kobayashi, M. *et al*. Molecular evolution of the capsid gene in human norovirus genogroup II. *Sci. Rep.* **6**, 29400; doi: 10.1038/srep29400 (2016).

## Supplementary Material

Supplementary Information

## Figures and Tables

**Table 1:** Positive selection sites on capsid gene in human NoV GII.

Amino acid change	SLAC	FEL	IFEL	MEME
Ser6AsnAsn6Ser, Lys, Ile	○	○	○	○
Asn9Thr, SerThr9AlaAla9ThrSer9AsnAsn9Thr,Lys,SerAla9Val, Thr		○		○
Thr16Ala, SerAla16Ser, Thr				○
Val23Ile, AlaIle23Val, Ser, AlaAla23Gly			○	
Asn25Ser, Thr, His, Gln,MixSer25Asn				○
Glu64Mix, His				○
Cys268Ser, Ala, ValVal268Cys, AlaSer268Thr				○
Asp297His, Asn, Ser, Gly, Val, GluHis297Pro, Gln, AspPro297SerGln297HisAsn297Ile, SerGly297Ser, Pro, Arg, AlaSer297Asn, Ala			○	
Gly298Asp, Arg, Ala, Ile, Gln, Asn, LysAsp298Gly, Asn, GluArg298Ser				○
Ala303Val, Ile, ThrThr303Val				○
Asp359AlaThr359SerAla359Ser, ValSer359Asn, GlyPro359ThrSer359Asn			○	○
Ala360Thr, Ser		○		
Gly370Ala, Ser, MixAla370Ser, Gly				○
Ser379Thr, Asp, Ala, Gly, Asn, ProAsp379AsnGly379Ser, AspAla379SerAsn379AspThr379Ser, Ala				○
Asn397Ser, Asp, Glu, Gly, Thr, GlnSer397Arg, AspGly397Ser, Asp,Asp397Glu, AsnHis397ArgThr397ProGln397Asp				○
Gly416Asp, Ala, SerAsp416Gly, Ser, Asn, GluGlu416AspHis416Asn, Gln, ArgAsn416Arg, Thr, AspThr416Pro, AlaSer416Thr				○
Asp416Asn, Gln, Gly, Ser, Glu, AlaAsn416Asp, Ser, GlySer416AlaGly416Ser				○
Ala419ThrThr419Asn, AlaAsn419Asp, AlaAsp419GlyThr419AlaAsp419Pro	○	○	○	○
Arg435Thr, HisThr435Pro, ValPro435His, SerHis435Ala, Arg, GlnAla435Arg, Ser, His, ValGln435Pro				○
Trp485Phe				○

mean *d*N/*d*S = 0.106 (95% CI = 0.103–0.109). Cut off *p*-value < 0.05.

**Table 2:** Predicted linear B-cell epitopes of standard strains for each genotype

Genotype	Strain (Accession No.)	Position	Predicted epitopes
GII.1	Hawaii virus/1971/US (U07611)	305–326	VTNTNGTPF**DPTEDVPAPLG**TP
		357–366	PKFTPKLGSV
GII.2	Melksham/1994/UK (X81879)	4–15	AS**N**DA**A**PSTDG**A**
		313–326	FD**PSEDIPAPLG**VP
		359–373	VPTY**T**AQYTPKLGQI
		531–541	PMGTGNGRRRV
GII.3	Toronto24/1991/CA (U02030)	59–68	APGG**E**FTVSP
		294–307	T**SR**ASDQ**A**DTPTPR
		325–338	Y**DPAEDIPAPLG**TP
		387–400	FDPNQPTKFTPVGV
GII.4	Bristol/1993/UK (X76716)	64–74	FTVSPRNAPGE
		125–135	PPNFPTEGLSP
		251–263	TGPSSAFVVQPQN
		309–326	SNY**DPTEEIPAPLG**TPDF
		436–445	TMPGCSGYPN
GII.5	Hillingdon/1990/UK (AJ277607)	64–73	FTVSPKNSPG
		213–222	TYLVPPTVES
		313–327	F**DLTDDVPAPLG**VPD
		337–351	SQRNRGESNPANR**A**H
		374–385	WNTNDVENQPTK
		439–448	PLKGGFGNPA
GII.7	Leeds/1990/UK (AJ277608)	306–328	ITNTDGTPI**DPTEDTPGPIG**SPD
		338–349	SQRNKNEQNPAT
		358–368	TGGDQYAPKLA
		390–401	VGVAGD**PS**HPFR
GII.8	Amsterdam/98-18/1998/NET (AF195848)	59–72	APAG**E**FTVSPRNAP
		308–327	NLDGSPV**DPTDEVPAPLG**TP
		369–383	FKSPSTDFSDNEPIK
GII.9	VA97207/1997/USA (AY038599)	4–15	AS**N**DA**A**PSSDG**A**
		59–72	APAG**E**FTVSPRNAP
		308–326	LDGSPI**DPTDDTPGPLG**CP
		336–380	ASQRGPGDATR**A**HEARIDTG**S**DTFAPKIGQVRFYSTS**S**DFETNQP
GII.10	Erfurt/546/2000/DE (AF427118)	4–15	AS**N**DA**A**PSSDG**A**
		204–223	TRPTPDFDFTYLVPPTVESK
		295–304	QDEHRG**T**HWN
		310–329	LNGTPF**DPTEDVPAPLG**TPD
		340–353	QRNTNT**VP**GEGDLP
		384–396	**Q**DVSSGQPTKFTP
GII.13	Fayetteville/1998/US (AY113106)	217–230	PPSVESKTKPFTLP
		250–263	YTAPNETNVVQCQN
		308–325	PNGASY**DPTDEVPAPLG**T
GII.14	M7/1999/US (AY130761)	307–325	LDGSPI**DPTDDMPAPLG**TP
		363-385	IGQVRFKSSS**D**DFDLHDPTKFTP
		455–466	EHFYQEAAPSQS
GII.15	J23/1999/US (AY130762)	20–30	VPE**S**Q**Q**EVLPL
		316–336	EPDGEEF**SPTGPNPAPVG**TPD
		349–359	NTGGAGQNSNR
		427–440	AGKLAPPVAPNYPG
GII.16	Tiffin/1999/USA (AY502010)	310–325	GTPF**DPTDDVPAPLG**M
		338–349	QRDTGTNPANR**A**
		359–378	AKYTPKLGSVQIGTWD**TE**DL
		380–389	ERQPVKFTPV
		434–447	FRSYIPLKGGHGDP
GII.17	CS-E1/2002/USA (AY502009)	7–17	DA**A**PSNDG**A**TG
		314–328	F**DPTEDVPAPLG**TPD
		341–351	NVGSNPNTTR**A**
		365–379	PKLGSVNFGSTSTDF
		420–432	PPIAPNFPGEQLL
GII.20	Luckenwalde591/2002/DE (EU373815)	59-69	APGG**E**FTVSPR
		125–135	PPNFPPENLSP
		308–323	NGSAY**DPTEDIPAVLG**
		337–346	QRSPNNSTR**A**
		350–361	TLNTGSPRYTPK
GII.21	IF1998/2003/IR (AY675554)	2–12	AS**K**DA**A**PSNDG
		211–222	TYLVPPSVESKT
		248–261	YTSPNADVVVQPQN
		310–323	TY**DPTEDVPAPFG**T
		335–348	TQNPRASGDEAAN**S**
		374–384	GHHSQHQQSKF
		457–468	HFYQESAPSQSD
GII.22	YURI2002/JP (AB083780)	159–172	PDVRNQFFHYNQVN
		217–226	PPTVESRTKP
		315–328	**DPTEDVPAPLG**TPD
		341–369	NDYN**D**GSQGPANR**A**HDAVVPTT**S**AKFTPK
		441–450	LKGGHGNPAI

Linear epitopes of GII.6 and GII.12 could not be predicted. The positions of the amino acids correspond to each strain. Common epitopes sequences are shown in the bold letters. Positive selection sites are shown in underlined text.
