# Supplementary material for: Molecular evolution of the capsid gene in human norovirus genogroup II
Source: Sci Rep. 2016 Jul 7;6:29400. doi: 10.1038/srep29400 (PMC4935990; doi:10.1038/srep29400)
Supplement: Supplementary Information [file srep29400-s1.pdf]

Molecular evolution of the capsid gene in human norovirus genogroup II

Miho Kobayashi, Yuki Matsushima, Takumi Motoya, Naomi Sakon, Naoki Shigemoto, Reiko Okamoto-Nakagawa, Koichi Nishimura, Yasutaka Yamashita, Makoto Kuroda, Nobuhiro Saruki, Akihide Ryo, Takeshi Saraya, Yukio Morita, Komei Shirabe, Mariko Ishikawa, Tomoko Takahashi, Hiroto Shinomiya, Nobuhiko Okabe, Koo Nagasawa, Yoshiyuki Suzuki, Kazuhiko Katayama* and Hirokazu Kimura **

*E-mail: katayama@nih.go.jp

**E-mail: kimhiro@nih.go.jp

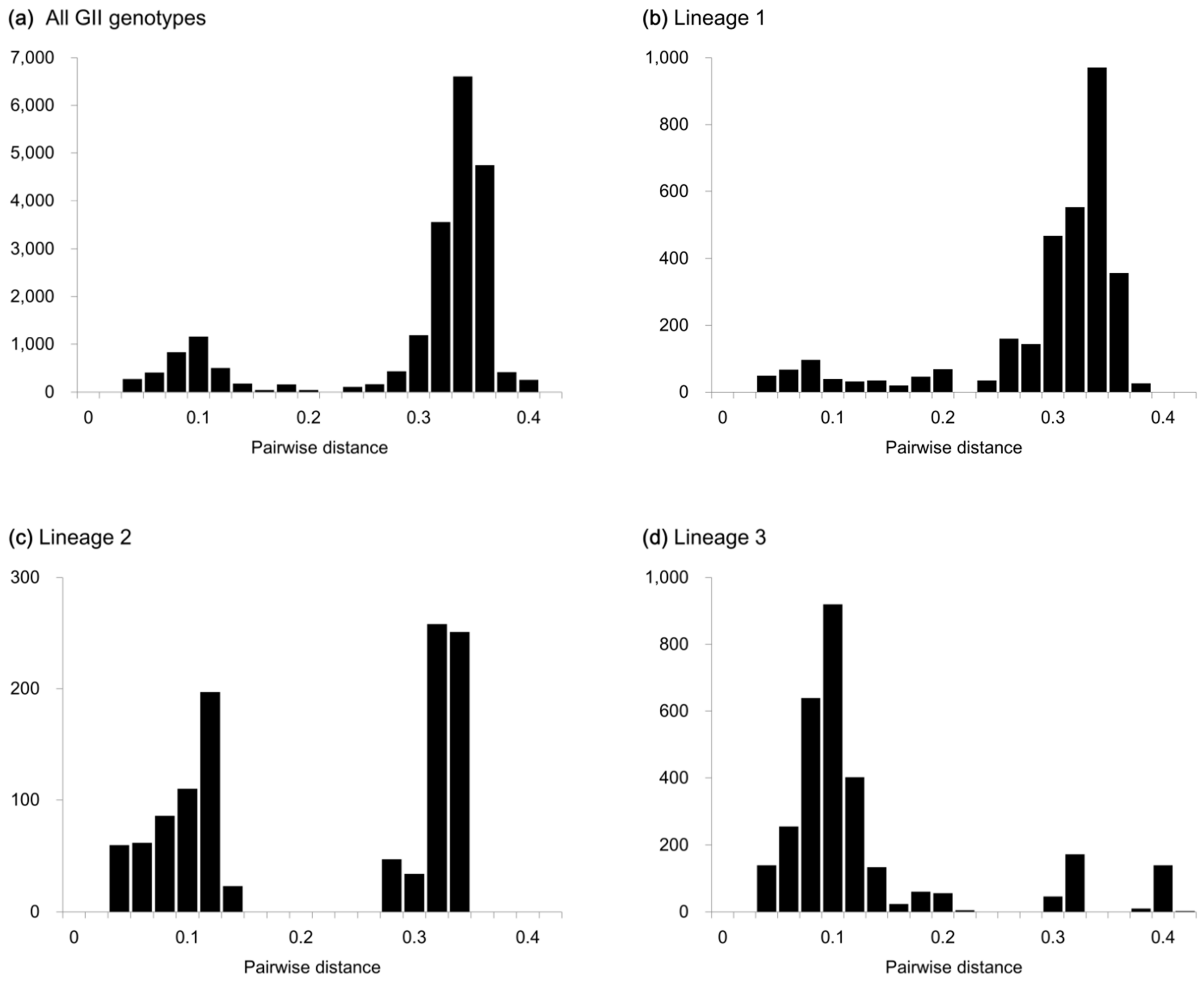

Supplementary Figure S1. Distribution of pairwise distances based on the nucleotide sequences of capsid gene among intergenogroups (**a**), lineage 1 (**b**), lineage 2 (**c**), and lineage 3 (**d**). The x-axis shows the pairwise distance, and the y-axis shows the number of pairs.

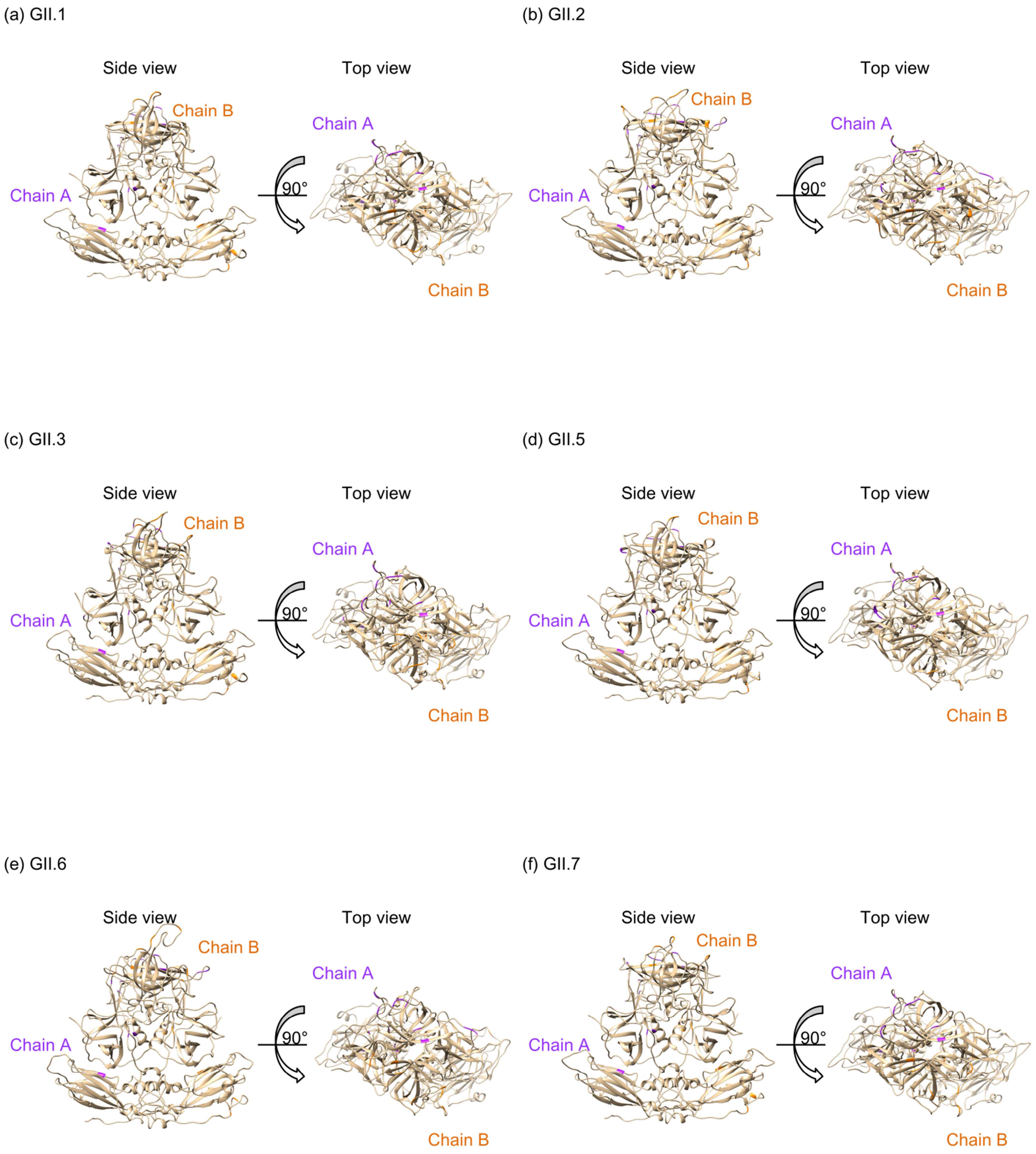
(a) GII.1
Side view
Top view
Chain A
Chain B
90°
(b) GII.2
Side view
Top view
Chain A
Chain B
90°
(c) GII.3
Side view
Top view
Chain A
Chain B
90°
(d) GII.5
Side view
Top view
Chain A
Chain B
90°
(e) GII.6
Side view
Top view
Chain A
Chain B
90°
(f) GII.7
Side view
Top view
Chain A
Chain B
90°

(g) GII.8

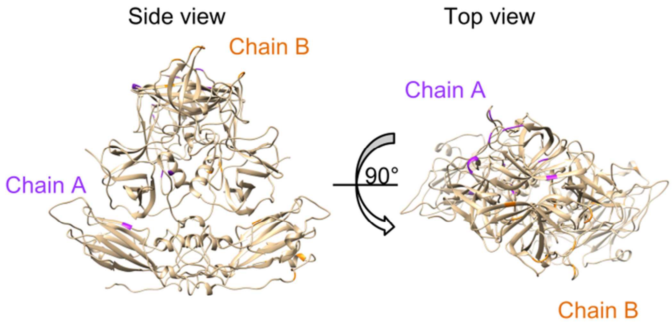

(h) GII.9

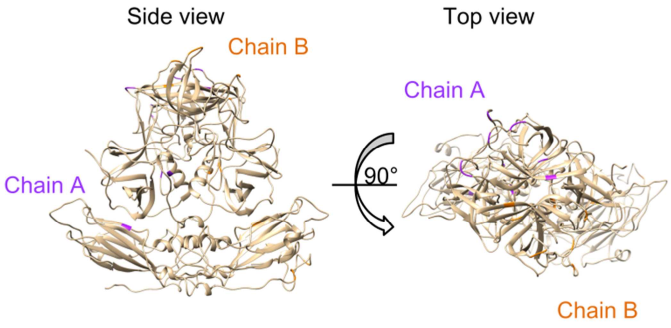

(i) GII.10

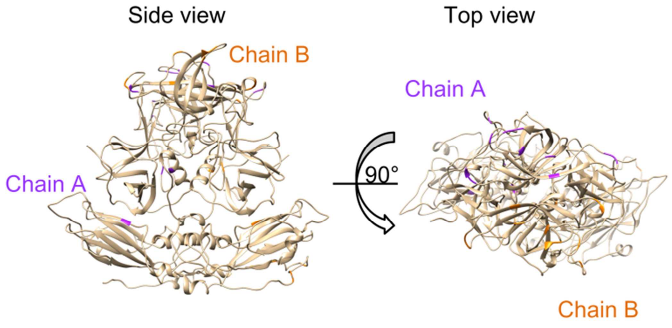

(j) GII.12

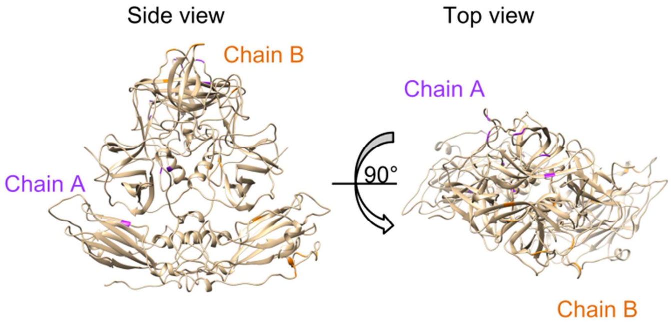

(k) GII.13

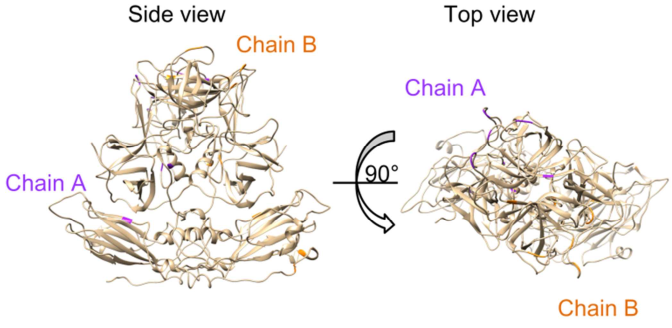

(l) GII.14

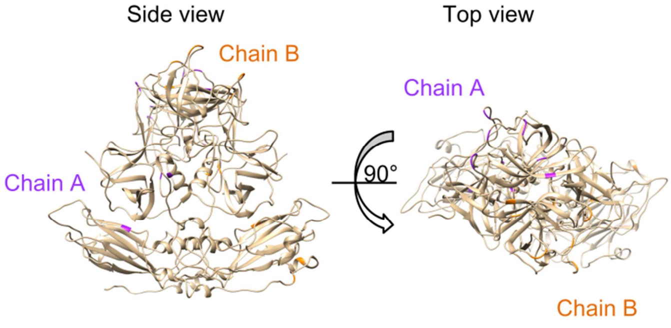

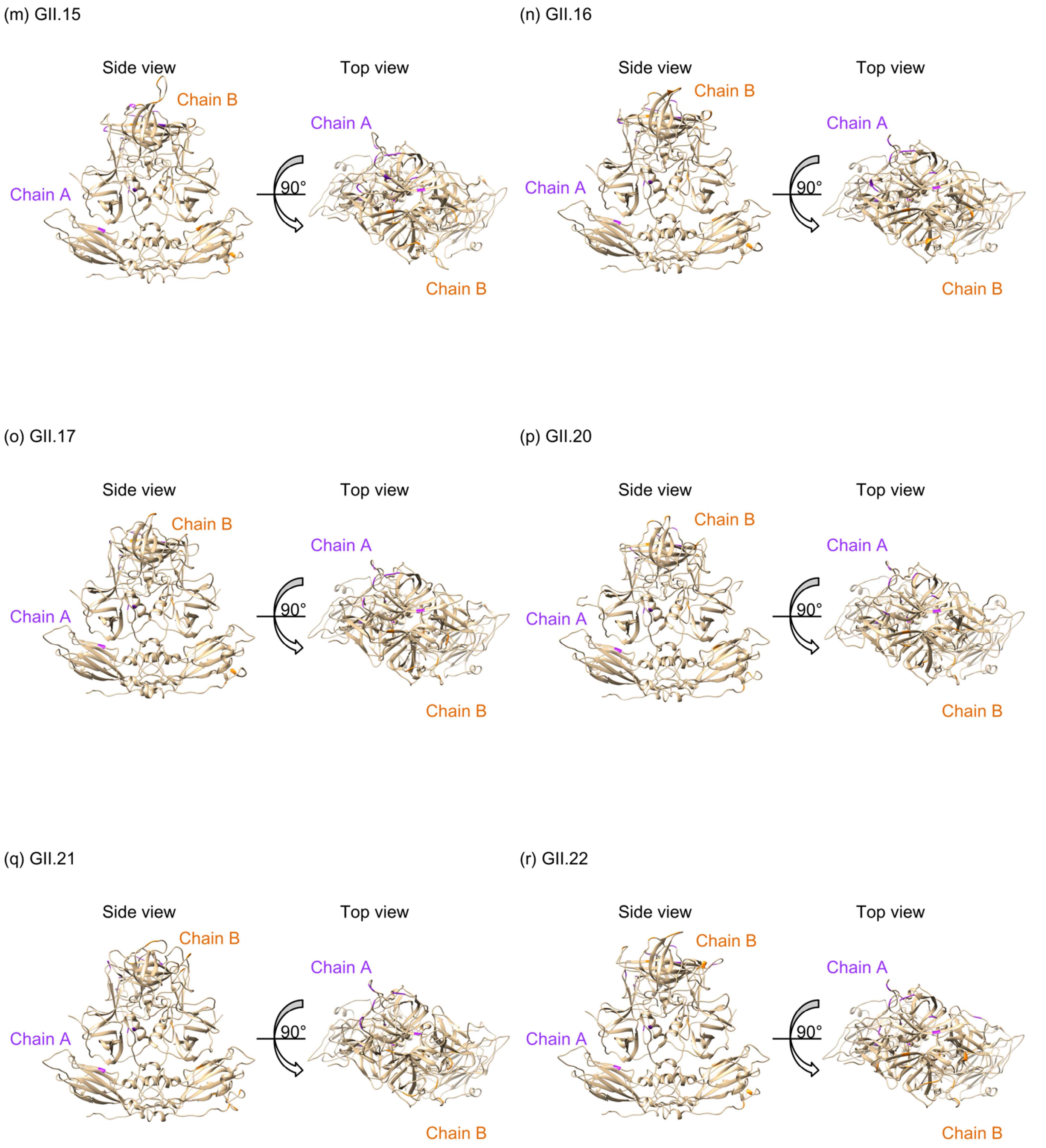

Supplementary Figure S2. Location of positive selection sites on the predicted structures of capsid protein in each genotype. Positive selection sites on chains A and B are colored purple and orange, respectively.

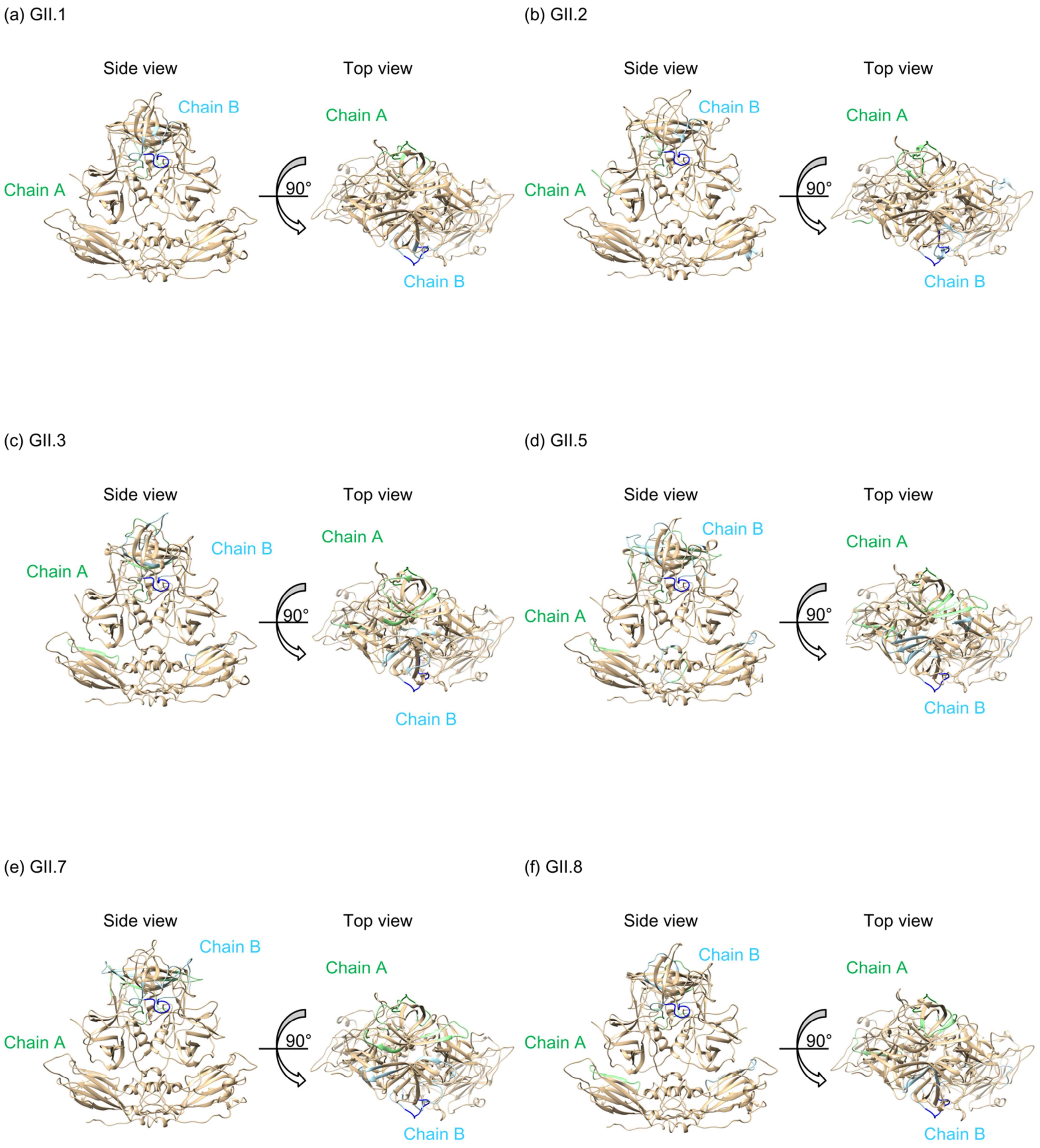
(a) GII.1
Side view
Top view
Chain B
Chain A
Chain A
90°
Chain B
(b) GII.2
Side view
Top view
Chain B
Chain A
Chain A
90°
Chain B
(c) GII.3
Side view
Top view
Chain A
Chain B
Chain A
90°
Chain B
(d) GII.5
Side view
Top view
Chain B
Chain A
Chain A
90°
Chain B
(e) GII.7
Side view
Top view
Chain B
Chain A
Chain A
90°
Chain B
(f) GII.8
Side view
Top view
Chain B
Chain A
Chain A
90°
Chain B

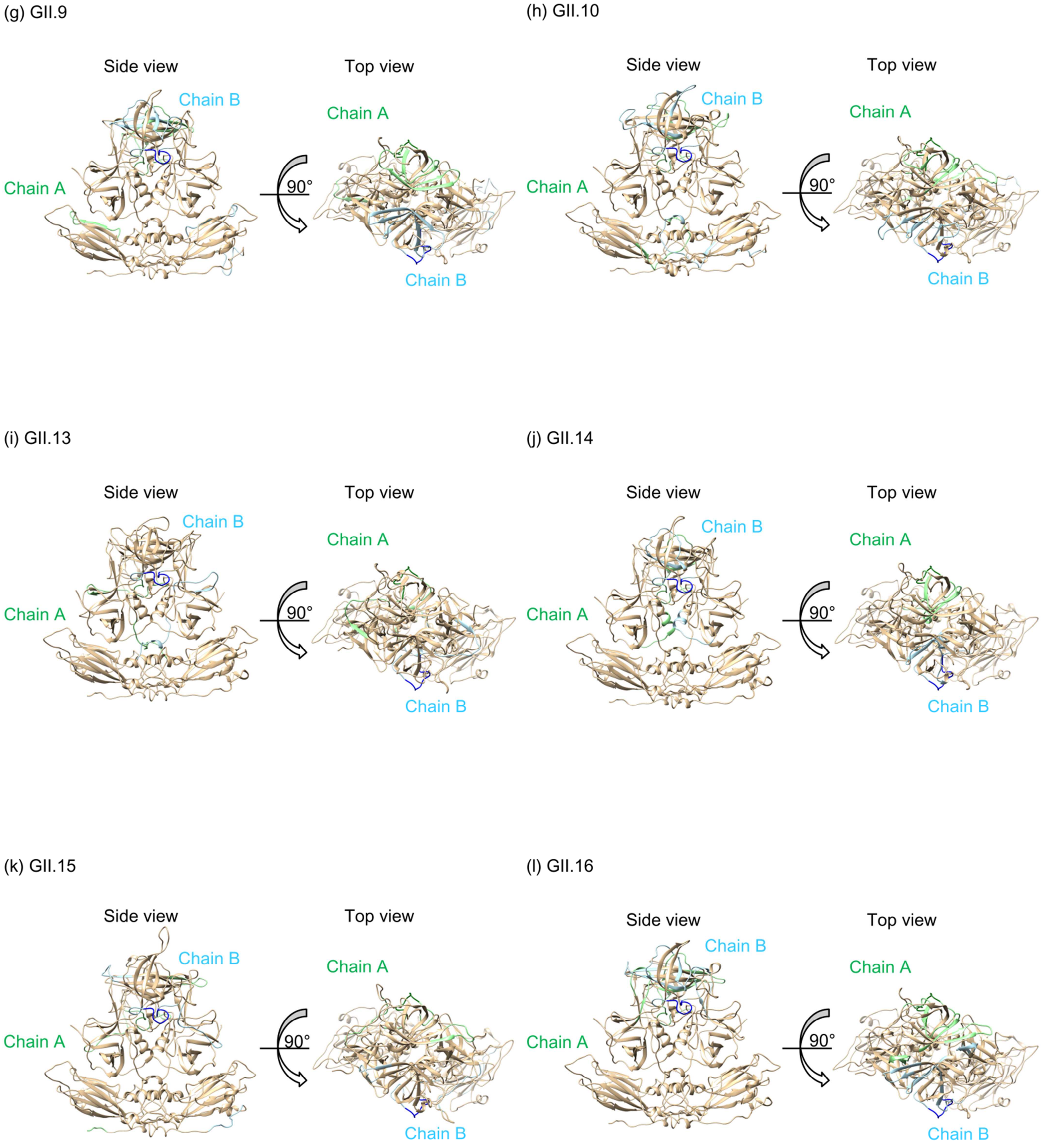
(g) GII.9
Side view
Top view
Chain B
Chain A
Chain A
90°
Chain B
(h) GII.10
Side view
Top view
Chain B
Chain A
Chain A
90°
Chain B
(i) GII.13
Side view
Top view
Chain B
Chain A
Chain A
90°
Chain B
(j) GII.14
Side view
Top view
Chain B
Chain A
Chain A
90°
Chain B
(k) GII.15
Side view
Top view
Chain B
Chain A
Chain A
90°
Chain B
(l) GII.16
Side view
Top view
Chain B
Chain A
Chain A
90°
Chain B

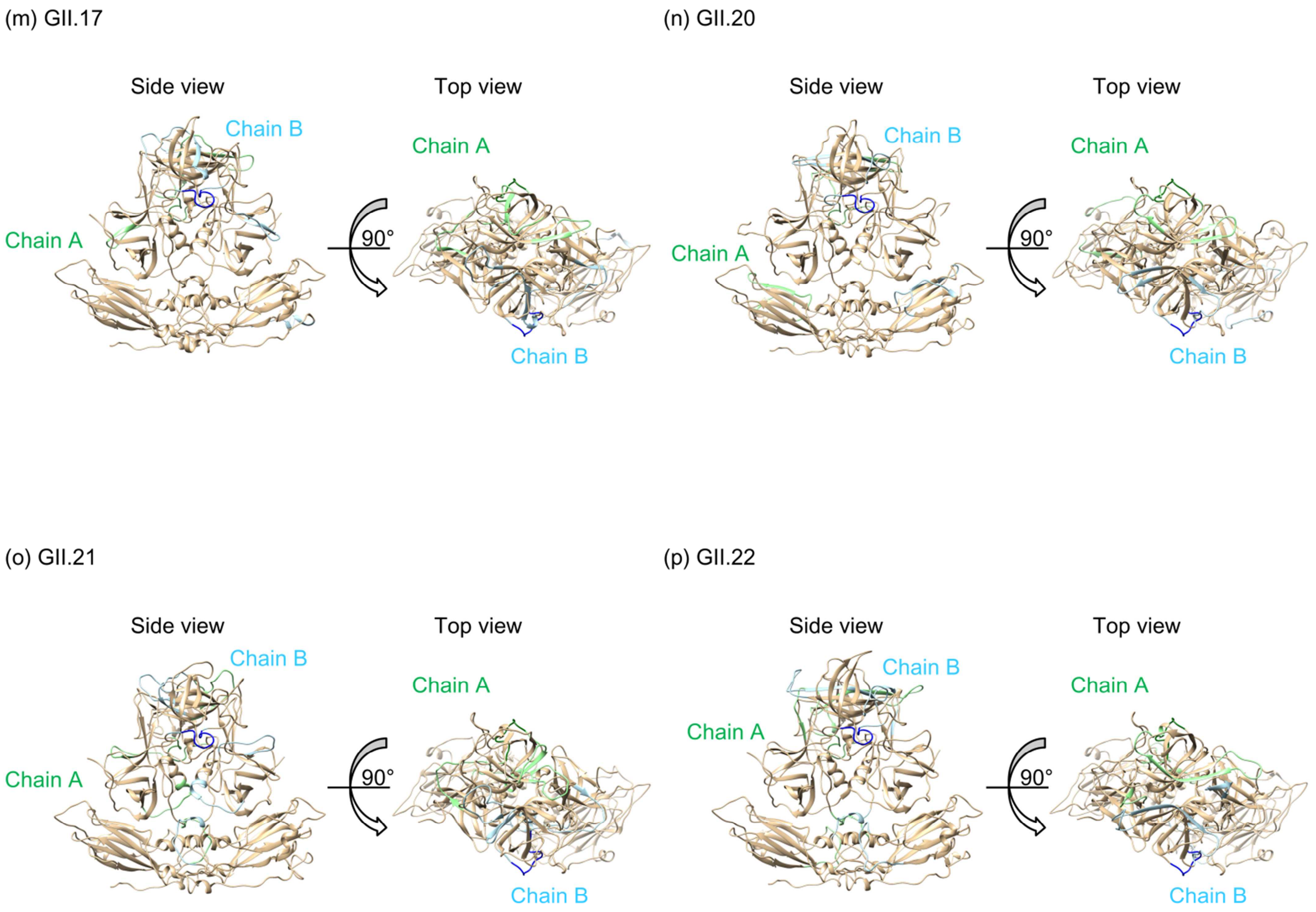

Supplementary Figure S3. Predicted linear B-cell epitopes mapping on the capsid proteins of each genotype. Linear B-cell epitopes on chains A and B are shown in green and blue, respectively. Common locations among all genotypes are represented by deeper tones.

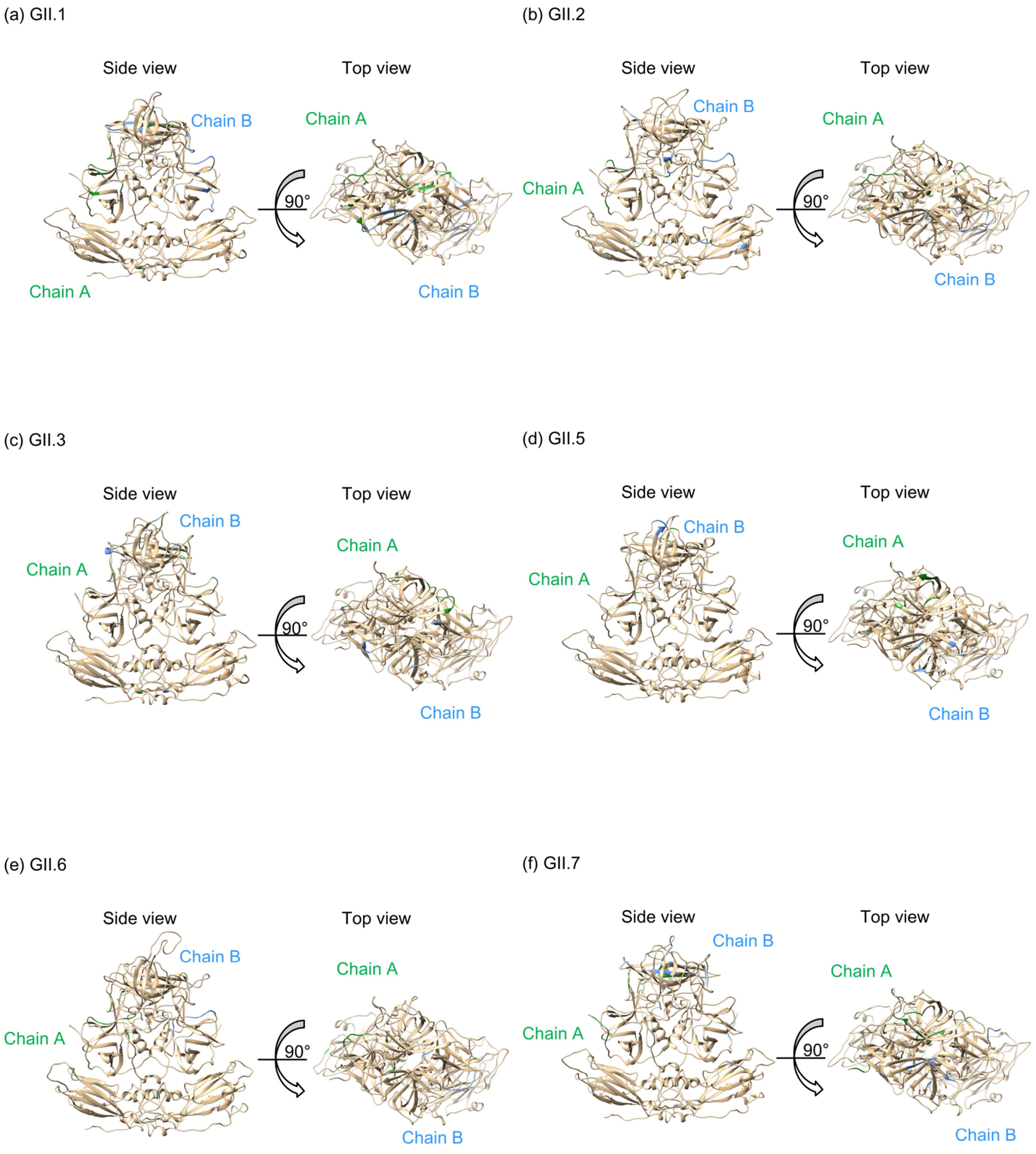
(a) GII.1
Side view
Top view
Chain B
Chain A
90°
Chain A
Chain B
(b) GII.2
Side view
Top view
Chain B
Chain A
Chain A
90°
Chain B
(c) GII.3
Side view
Top view
Chain B
Chain A
Chain A
90°
Chain B
(d) GII.5
Side view
Top view
Chain B
Chain A
Chain A
90°
Chain B
(e) GII.6
Side view
Top view
Chain B
Chain A
Chain A
90°
Chain B
(f) GII.7
Side view
Top view
Chain B
Chain A
Chain A
90°
Chain B

(g) GII.8

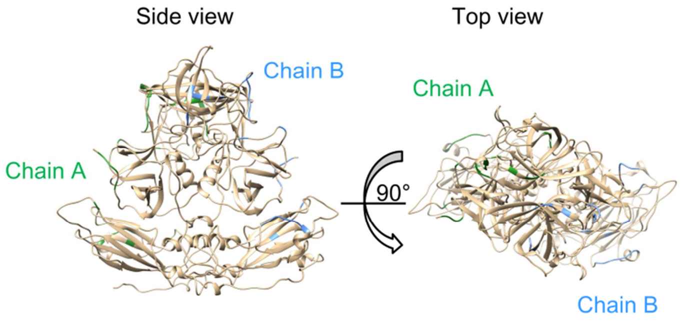

(h) GII.9

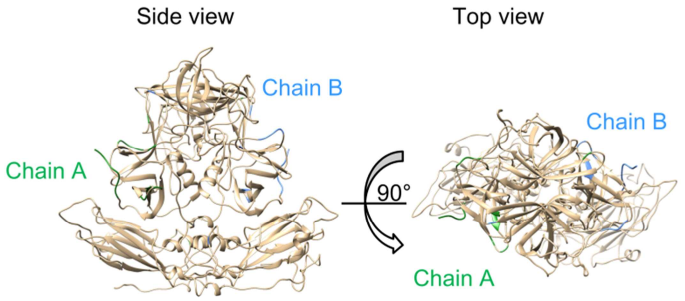

(i) GII.10

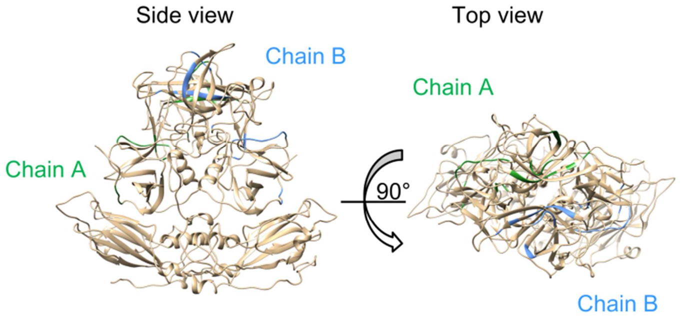

(j) GII.12

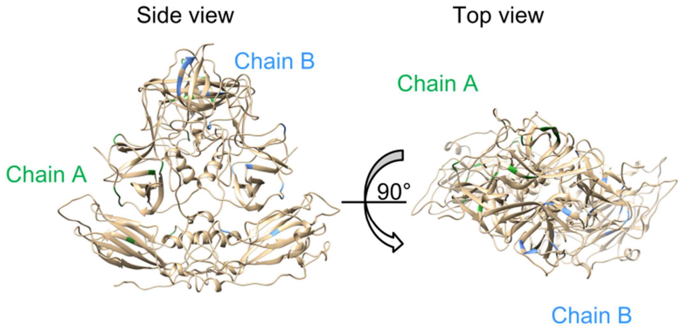

(k) GII.13

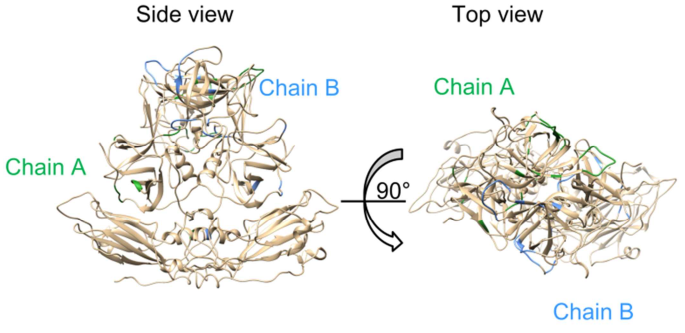

(l) GII.14

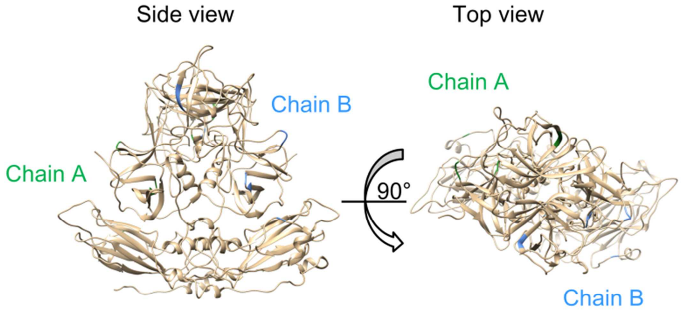

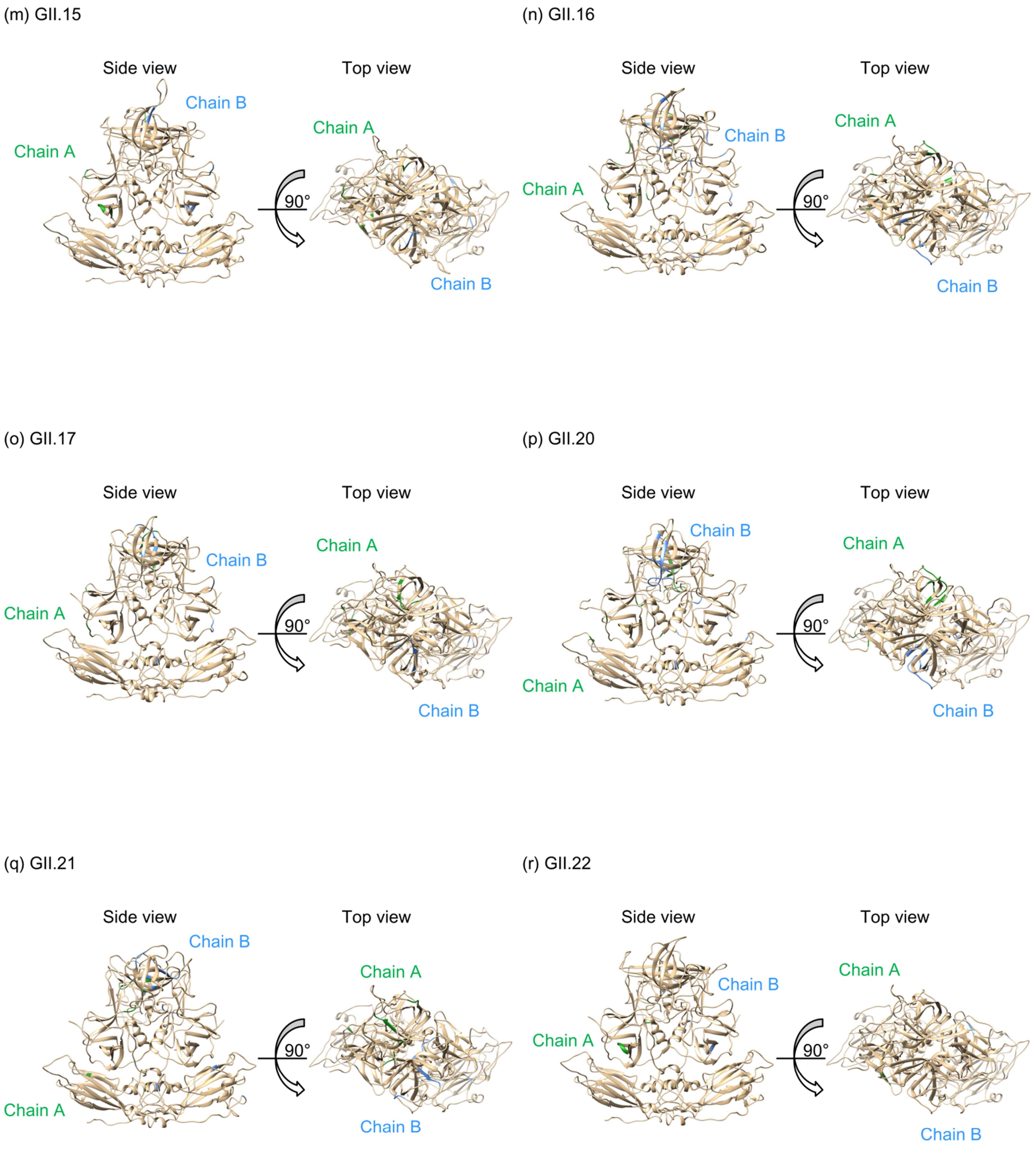

Supplementary Figure S4. Predicted conformational B-cell epitopes mapping on the capsid proteins of each genotype. The predicted epitopes of chains A and B are shown in green and blue, respectively.

Supplementary Table S1 Year of divergence of each genotype in NoV GII

Genogroup	Lineage	Genotype	Year of divergence (95% HPDs)
GII	1	GII.1,2,5,6,10,11,12,13,16,17,18,19,21,22	1819 (1755−1876)
	2	GII.3,7,8,9,14	1839 (1773−1903)
	3	GII.4,15,20	1630 (1409−1796)
		GII.1	1957 (1945−1967)
		GII.2	1960 (1945−1973)
		GII.3	1946 (1918−1970)
		GII.4	1924 (1889−1952)
		GII.5	2000 (1958−1977)
		GII.6	1928 (1895−1956)
		GII.7	1956 (1921−1972)
		GII.8	1948 (1921−1970)
		GII.9	1978 (1970−1983)
		GII.10	1918 (1888−1944)
		GII.11	1964 (1934−1986)
		GII.12	1972 (1956−1984)
		GII.13	1975 (1964−1982)
		GII.14	1967 (1953−1977)
		GII.15	1994 (1983−1999)
		GII.16	1989 (1978−1997)
		GII.17	1950 (1922−1971)
		GII.18	1913 (1861−1960)
		GII.19	1964 (1934−1986)
		GII.20	1988 (1971−2000)
		GII.21	1987 (1971−1998)
		GII.22	1975 (1942−1997)
GI			1761 (1576−1900)
GIII			1444 (978−1784)
GIV			1985 (1969−1995)

HPDs, highest posterior densities

Supplementary Table S2 Predicted conformational B-cell epitopes of standard strains for each genotype

Genotype	No. of sites detected as conformational epitopes
GII.1	26
GII.2	20
GII.3	12
GII.4	28
GII.5	12
GII.6	16
GII.7	18
GII.8	27
GII.9	20
GII.10	30
GII.12	20
GII.13	36
GII.14	11
GII.15	11
GII.16	17
GII.17	11
GII.20	24
GII.21	16
GII.22	4

SVM threshold was set at -0.0.

Supplementary Table S3 Strains used in this study

GenBank accession	Genotype	Name
**U07611**	**GII.1**	**Hu/GII.1/Hawaii virus/1971/US**
AF414416	GII.1	Hu/GII.1/Miami/81/1986/US
AF414418	GII.1	Hu/GII.1/Westover/302/1994/US
AF425767	GII.1	Hu/GII.1/Dillingen 391/2001/DE
AJ277606	GII.1	Hu/GII.1/Girlington/1993/UK
EF547397	GII.1	Hu/GII.1/Saga/001215/3101/2000/JP
EU072249	GII.1	Hu/GII.1/CHN41808/LL04/2004/CHN
JN616378	GII.1	Hu/GII.1/Kazincbarcika/HUN4593/2010/HUN
JN797508	GII.1	Hu/GII.1/Ascension208/2010/USA
KJ194507	GII.1	Hu/GII.1/Amsterdam/3/1995/DNK
**X81879**	**GII.2**	**Hu/GII.2/Melksham/1994/UK**
AB195225	GII.2	Hu/GII.2/Ina/2002/JP
AB279554	GII.2	Hu/GII.2/OC01243/2001/JP
AB279557	GII.2	Hu/GII.2/OC04038/2004/JP
AB279569	GII.2	Hu/GII.2/OC05010/2005/JP
AB279570	GII.2	Hu/GII.2/OCS020289/2002/JP
AB281081	GII.2	Hu/GII.2/Coevorden191S/1999/NL
AB281082	GII.2	Hu/GII.2/DenHaag37/2000/NL
AB281084	GII.2	Hu/GII.2/Leeuwarden15/2001/NL
AB281085	GII.2	Hu/GII.2/Zwolle25E/2001/NL
AB281088	GII.2	Hu/GII.2/Leeuwarden71/2003/NL
AB535749	GII.2	Hu/GII.2/OC080306/2008/JP
AB629946	GII.2	Hu/GII.2/Tokyo/10-4320/2011/JP
AB662859	GII.2	Hu/GII.2/OC08079/2008/JP
AB662861	GII.2	Hu/GII.2/OC08154/2008/JP
AB662870	GII.2	Hu/GII.2/OC09072/2009/JP
AY054300	GII.2	Hu/GII.2/Chesterfield/434/1997/US
EF547398	GII.2	Hu/GII.2/Maizuru/000602/2840/2000/JP
JN699037	GII.2	Hu/GII.2/KL109/1978/MY
JQ320072	GII.2	Hu/GII.2/NF2002/2002/USA
**U02030**	**GII.3**	**Hu/GII.3/Toronto24/1991/CA**
AB195226	GII.3	Hu/GII.3/Sinsiro/1997/JP
AB385634	GII.3	Hu/GII.3/RotterdamP5D0/2005/NL
AB758450	GII.3	Hu/GII.3/Miyagi/2009/JP
AF414413	GII.3	Hu/GII.3/Montgomery/312/1994/US
AF427111	GII.3	Hu/GII.3/Berlin/226/2001/DE
AF539439	GII.3	Hu/GII.3/Herzberg 385/2001/DE
AJ277611	GII.3	Hu/GII.3/Bham132/1995/UK
AY247431	GII.3	Hu/GII.3/1937-00/2000/SWE
AY652979	GII.3	Hu/GII.3/Paris Island/2003/USA
AY845056	GII.3	Hu/GII.3/C14/2002/AU
DQ379713	GII.3	Hu/GII.3/Goulburn Valley G5175 A/1983/AUS
EF547399	GII.3	Hu/GII.3/Maizuru/010524/3229/2001/JP
EU072243	GII.3	Hu/GII.3/CHN39246/CC04/2004/CHN
EU921389	GII.3	Hu/GII.3/Pune/PC52/2007/India
GQ849127	GII.3	Hu/GII.3/Sydney740C/2007/AUS
GQ856467	GII.3	Hu/GII.3/Beijing/55040/2007/CHN
GU991355	GII.3	Hu/GII.3/Shanghai/SH312/2009/CHN
HM072041	GII.3	Hu/GII.3/CHDC5261/1990/US
HM072042	GII.3	Hu/GII.3/CHDC4671/1979/US
HM072043	GII.3	Hu/GII.3/CHDC4090/1988/US
HM072045	GII.3	Hu/GII.3/CHDC2005/1975/US
JN565063	GII.3	Hu/GII.3/Milwaukee009/2010/USA
JN699040	GII.3	Hu/GII.3/HK54/1977/CN

GenBank accession	Genotype	Name
JQ743333	GII.3	Hu/GII.3/1999/USA
JX984948	GII.3	Hu/GII.3/GZ2010-L63/Guangzhou/2010/CHN
KC597140	GII.3	Hu/GII.3/NIHIC8.1/2011/USA
KF306213	GII.3	Hu/GII.3/Jingzhou/2013402/2013/CHN
KJ194500	GII.3	Hu/GII.3/Amsterdam/1/1995/DNK
KJ194504	GII.3	Hu/GII.3/Amsterdam/1994/DNK
KJ499441	GII.3	Hu/GII.3/CUHK-NS-193/2013/HKG
KJ499443	GII.3	Hu/GII.3/CUHK-NS-218/2013/HKG
GU270580	**GII.4 (Apeldoorn 2007)**	**Hu/GII.4/New Orleans1500/2008/USA**
HM635101	GII.4 (Apeldoorn 2007)	Hu/GII.4/Seoul/0921/2009/KOR
HM635155	GII.4 (Apeldoorn 2007)	Hu/GII.4/Seoul/0902/2009/KOR
KC409311	GII.4 (Apeldoorn 2007)	Hu/GII.4/30199/2009/VNM
KF429782	GII.4 (Apeldoorn 2007)	Hu/GII.4/NIHIC1.3/2010/USA
AB220921	**GII.4 (Asia2003)**	**Hu/GII.4/Chiba/04-1050/2005/JP**
X76716	**GII.4 (Bristol 1993)**	**Hu/GII.4/Bristol/1993/UK**
FJ537137	GII.4 (Bristol 1993)	Hu/GII.4/CHDC4108/1987/US
AF145896	**GII.4 (Camberwell 1994)**	**Hu/GII.4/Camberwell/101922/1994/AUS**
AY030098	**GII.4 (Camberwell 1994)**	**Hu/GII.4/MD134-7/1987/US**
FJ537136	GII.4 (Camberwell 1994)	Hu/GII.4/CHDC3967/1988/US
JX289821	GII.4 (Camberwell 1994)	Hu/GII.4/MD120-12/1987/USA
AB541362	GII.4	Hu/GII.4/Toyama5/2008/JP
EU078406	GII.4	Hu/GII.4/Richmond/1994/USA
EU078416	GII.4	Hu/GII.4/Billings/2006/USA
FJ537134	GII.4	Hu/GII.4/CHDC5191/1974/US
FJ537135	GII.4	Hu/GII.4/CHDC2094/1974/US
HQ008054	GII.4	Hu/GII.4/2405/2008/ZAF
HQ008055	GII.4	Hu/GII.4/8483/2008/ZAF
HQ008056	GII.4	Hu/GII.4/4638/2008/ZAF
JX401281	GII.4	Hu/GII.4/T091/1976/TN
KC576915	GII.4	Hu/GII.4/KL45/1978/MYS
KF429760	GII.4	Hu/GII.4/NIHIC28.4/2012/USA
KF429768	GII.4	Hu/GII.4/NIHIC1.13/2012/USA
EF126965	**GII.4 (Den Haag 2006b)**	**Hu/GII.4/DenHaag89/2006/NL**
AB541246	GII.4 (Den Haag 2006b)	Hu/GII.4/Fukui2/2008/JP
AB541254	GII.4 (Den Haag 2006b)	Hu/GII.4/Hiroshima2/2008/JP
JF713049	GII.4 (Den Haag 2006b)	Hu/GII.4/Hong Kong/CUB001-September/2010/CHN
JQ613562	GII.4 (Den Haag 2006b)	Hu/GII.4/NSW827D/2010/AU
JQ613572	GII.4 (Den Haag 2006b)	Hu/GII.4/StVincents/NSW217I/2010/AU
JX459648	GII.4 (Den Haag 2006b)	Hu/GII.4/SG4058-10/2009/SG
JX459905	GII.4 (Den Haag 2006b)	Hu/GII.4/Randwick/NSW938K/2011/AU
JX984947	GII.4 (Den Haag 2006b)	Hu/GII.4/GZ2010-L32/Guangzhou/2010/CHN
KC576909	GII.4 (Den Haag 2006b)	Hu/GII.4/NIHIC4.2/2011/USA
KF712496	GII.4 (Den Haag 2006b)	Hu/GII.4/NIHIC2.3/2010/USA
AY485642	**GII.4 (Farmington Hills 2002)**	**Hu/GII.4/Langen1061/2002/DE**
AB303936	GII.4 (Farmington Hills 2002)	Hu/GII.4/Elsloo012/2004/NL
EU078408	GII.4 (Farmington Hills 2002)	Hu/GII.4/Lonaconing/2001/USA(FAMINGSTO
AY883096	**GII.4 (Hunter 2004)**	**Hu/GII.4/2004/NL**
HQ008057	GII.4 (Hunter 2004)	Hu/GII.4/5467/2008/ZAF
JX459599	GII.4 (Hunter 2004)	Hu/GII.4/SG4091-06/2006/SG
AB303929	**GII.4 (Kaiso2003)**	**Hu/GII.4/EmmenE006/2002/NL**
AB186065	GII.4 (Kaiso2003)	Hu/GII.4/OC02202/2002/JP
DQ364459	**GII.4 (Lanzou 2002)**	**Hu/GII.4/Lanzhou/35666/2002/China**
EU310927	**GII.4 (Lanzou 2002)**	**Hu/GII.4/Houston/TCH186/2002/US**
EU078412	GII.4 (Lanzou 2002)	Hu/GII.4/FL04/2004/USA

GenBank accession	Genotype	Name
GU445325	**GII.4 (New Orleans 2009)**	**Hu/GII.4/New Orleans1805/2009/USA**
HF952120	GII.4 (New Orleans 2009)	Hu/GII.4/C00007876/2011/UK
JQ613564	GII.4 (New Orleans 2009)	Hu/GII.4/NSW944J/2010/AU
KF429762	GII.4 (New Orleans 2009)	Hu/GII.4/NIHIC17.7/2012/USA
KF712502	GII.4 (New Orleans 2009)	Hu/GII.4/NIHIC35/2013/USA
KF712504	GII.4 (New Orleans 2009)	Hu/GII.4/NIHIC11.3/2013/USA
AB434770	**GII.4 (Osaka 2007)**	**Hu/GII.4/OC07138/2007/JP**
EU876882	GII.4 (Osaka 2007)	Hu/GII.4/Cairo2/2006/EGY
EU876888	GII.4 (Osaka 2007)	Hu/GII.4/Cairo8/2007/EGY
FJ411171	GII.4 (Osaka 2007)	Hu/GII.4/SSCS/2005/USA
GQ246791	GII.4 (Osaka 2007)	Hu/GII.4/Dijon/E3020/2008/FRA
GQ413969	GII.4 (Osaka 2007)	Hu/GII.4/Riviera1635/2008/US
JX459908	**GII.4 (Sydney 2012)**	**Hu/GII.4/Sydney/NSW0514/2012/AU**
KF060122	GII.4 (Sydney 2012)	Hu/GII.4/NLV-10-603/2010/NZ
KF509947	GII.4 (Sydney 2012)	Hu/GII.4/AlbertaEI337/2011/CA
AJ004864	**GII.4 (US95 96)**	**Hu/GII.4/Grimsby/1995/UK**
AF080558	GII.4 (US95 96)	Hu/GII.4/408/97003012/1996/FL
AB504306	GII.4 (US95 96)	Hu/GII.4/Hiroshima/19/2001/JP
AF427115	GII.4 (US95 96)	Hu/GII.4/Ludwigslust/218/1999/DE
AF427117	GII.4 (US95 96)	Hu/GII.4/Erfurt/007/2000/DE
AF427120	GII.4 (US95 96)	Hu/GII.4/Beeskow/124/2000/DE
AY532118	GII.4 (US95 96)	Hu/GII.4/Dresden319/1997/GE
AY532132	GII.4 (US95 96)	Hu/GII.4/Hamburg180/1997/GE
EU078410	GII.4 (US95 96)	Hu/GII.4/GCanyon/2002/USA
JQ478407	GII.4 (US95 96)	Hu/GII.4/1997/USA
EF126963	**GII.4 (Yerseke 2006a)**	**Hu/GII.4/Yerseke38/2006/NL**
EF187497	GII.4 (Yerseke 2006a)	Hu/GII.4/Kenepuru/NZ327/2006/NZL
AB212306	GII.5	Hu/GII.5/Hokkaido/133/2003/JP
AF397156	GII.5	Hu/GII.5/1999/MOH
AJ277607	**GII.5**	**Hu/GII.5/Hillingdon/1990/UK**
JN699044	GII.5	Hu/GII.5/C15/1978/GF
AB682736	GII.6	Hu/GII.6/Ehime090371/2009/JP
AB685738	GII.6	Hu/GII.6/OC08025VLP/2008/JP
AB818400	GII.6	Hu/GII.6/Ehime120246/2012/JP
AB818402	GII.6	Hu/GII.6/Ehime041525/2004/JP
AB818403	GII.6	Hu/GII.6/Ehime031163/2003/JP
AB818404	GII.6	Hu/GII.6/Ehime030769/2003/JP
AF414407	GII.6	Hu/GII.6/Florida/269/1993/US
AF414408	GII.6	Hu/GII.6/Baltimore/274/1993/US
AF414410	GII.6	Hu/GII.6/Miami/292/1994/US
AJ277620	**GII.6**	**Hu/GII.6/Seacroft/1990/UK**
EF547401	GII.6	Hu/GII.6/Osaka/010203/3612/2001/JP
GU930737	GII.6	Hu/GII.6/E99-13646/1997/US
JN183165	GII.6	Hu/GII.6/S18/Lilla Edet/2008/Sweden
JN699035	GII.6	Hu/GII.6/S9c/1976/SN
JN699041	GII.6	Hu/GII.6/HK28/1977/CN
JX846927	GII.6	Hu/GII.6/CHDC4073/1984/USA
JX984945	GII.6	Hu/GII.6/GZ2010-L1/Guangzhou/2010/CHN
JX984953	GII.6	Hu/GII.6/GZ2010-L96/Guangzhou/2011/CHN
KC464321	GII.6	Hu/GII.6/Ohio/490/2012/USA
AF414409	GII.7	Hu/GII.7/Gwynedd/273/1994/US

GenBank accession	Genotype	Name
AJ277608	**GII.7**	**Hu/GII.7/Leeds/1990/UK**
EF547402	GII.7	Hu/GII.7/Kurume/951228/419/1995/JP
GQ849129	GII.7	Hu/GII.7/NSW088L/2007/AUS
GQ849130	GII.7	Hu/GII.7/NSW743L/2008/AUS
GU134965	GII.7	Hu/GII.7/1738/2009/USA
JN699042	GII.7	Hu/GII.7/HK4/1976/CN
KC832474	GII.7	Hu/GII.7/VLP 05G954/2005/DE
KF006266	GII.7	Hu/GII.7/TCH-134/2003/USA
AF195848	**GII.8**	**Hu/GII.8/Amsterdam/98-18/1998/NET**
AY038599	**GII.9**	**Hu/GII.9/VA97207/1997/USA**
AY054299	GII.9	Hu/GII.9/Idaho Falls/378/1996/US
DQ379715	GII.9	Hu/GII.9/Goulburn Valley G5175 C/1983/AUS
AF427118	**GII.10**	**Hu/GII.10/Erfurt/546/2000/DE**
AF414420	GII.12	Hu/GII.12/Honolulu/314/1994/US
AF427119	GII.12	Hu/GII.12/Pirna/110/2000/DE
AJ277618	**GII.12**	**Hu/GII.12/Wortley/1990/UK**
EU921353	GII.12	Hu/GII.12/Pune/PC24/2006/India
HQ115742	GII.12	Hu/GII.12/Velence/HUN4417/2010/HUN
KC464496	GII.12	Hu/GII.12/CGMH38/2010/TW
KF006267	GII.12	Hu/GII.12/Texas/E13842/2000/USA
AY113106	**GII.13**	**Hu/GII.13/Fayetteville/1998/US**
DQ379714	GII.13	Hu/GII.13/Goulburn Valley G5175 B/1983/AUS
EU921354	GII.13	Hu/GII.13/Pune/PC25/2006/India
JN899242	GII.13	Hu/GII.13/VA173/2010/USA
AY130761	**GII.14**	**Hu/GII.14/M7/1999/US**
EF670650	GII.14	Hu/GII.14/Shanxi/50106/2006/CHN
JN699038	GII.14	Hu/GII.14/HK74/1978/CN
AY130762	**GII.15**	**Hu/GII.15/J23/1999/US**
GQ856474	GII.15	Hu/GII.15/Beijing/55161/2008/CHN
AB195228	GII.16	Hu/GII.16/Kamo/2003/JP
AY502010	**GII.16**	**Hu/GII.16/Tiffin/1999/USA**
AY502011	GII.16	Hu/GII.16/Canton/1999/USA
EF547406	GII.16	Hu/GII.16/Osaka/010228/3625/2001/JP
AY502009	**GII.17**	**Hu/GII.17/CS-E1/2002/USA**
DQ438972	GII.17	Hu/GII.17/Katrina-17/2005/US
JN699043	GII.17	Hu/GII.17/C142/1978/GF
AB542917	GII.20	Hu/GII.20/OC07118/2007/JP
EU072235	GII.20	Hu/GII.20/CHN42973/CZ05/2005/CHN
EU373815	**GII.20**	**Hu/GII.20/Luckenwalde591/2002/DE**
AY675554	**GII.21**	**Hu/GII.21/IF1998/2003/IR**
AB542915	GII.21	Hu/GII.21/OC05024/2005/JP
EU019230	GII.21	Hu/GII.21/Ahm/PC03/2006/India
AB083780	**GII.22**	**Hu/GII.22/YURI/2002/JP**
GQ856469	GII.22	Hu/GII.22/Beijing/53931/2007/CHN
AB074893	GII.11	Sw/GII.11/Sw918/1997/JP
AY823304	GII.18	Sw/GII.18/OH-QW101/2003/US
AY823306	GII.19	Sw/GII.19/OH-QW170/2003/US
AJ011099	GIII.1	Bo/GIII.1/Jena/1980/DE
AF195847	GIV.1	Hu/GIV.1/Alphatron/98-2/1998/NET
AF414426	GIV.1	Hu/GIV.1/Fort Lauderdale/560/1998/US
AF414427	GIV.1	Hu/GIV.1/Saint Cloud/624/1998/US

GenBank accession	Genotype	Name
M87661	GI.1	Hu/GI.1/Norwalk/1968/US
L07418	GI.2	Hu/GI.2/Southampton/1991/UK
U04469	GI.3	Hu/GI.3/Desert Shield395/1990/UK
AB022679	GI.4	Hu/GI.4/Chiba 407/1987/JP
AJ277614	GI.5	Hu/GI.5/Musgrove/1989/UK
AF093797	GI.6	Hu/GI.6/BS5/1997/DE
AJ277609	GI.7	Hu/GI.7/Winchester/1994/UK
AF538679	GI.8	Hu/GI.8/Boxer/2001/US
HQ637267	GI.9	Hu/GI.9/Vancouver730/2004/CAN

Standard strains are shown as bold type.

Supplementary Table S4 Conditions of Bayesian skyline plot analysis

Genotype	No. of strains	Substitution model	Clock model	Length of MCMC chain
All genotypes of human GII	203	GTR-Γ	Exponential clock	2,000,000,000 (sampling every 20,000 steps)
GII.2	20	GTR-Γ	strict clock	30,000,000 (sampling every 10,000 steps)
GII.3	32	GTR-Γ	Exponential clock	40,000,000 (sampling every 10,000 steps)
GII.4	73	GTR-Γ	Exponential clock	100,000,000 (sampling every 10,000 steps)
GII.6	19	GTR-Γ	Exponential clock	50,000,000 (sampling every 10,000 steps)
